# Assessment of Carotid Plaque Stability Using Contrast-Enhanced Ultrasound and Its Correlation With the Expression of CD147 and MMP-9 in the Plaque

**DOI:** 10.3389/fncom.2021.778946

**Published:** 2021-12-01

**Authors:** Shanshan Huang, Xinyin Wu, Linlin Zhang, Jianming Wu, Yi He, Manlin Lai, Jiaqi Xu, Zhenzhou Li

**Affiliations:** ^1^Department of Ultrasound, Shenzhen Second People’s Hospital, The First Affiliated Hospital of Shenzhen University, Shenzhen, China; ^2^Department of Neurosurgery, Shenzhen Second People’s Hospital, The First Affiliated Hospital of Shenzhen University, Shenzhen, China

**Keywords:** carotid plaque, plaque, carotid endarterectomy, carotid stenosis, contrast-enhanced ultrasound

## Abstract

This study aims to investigate the correlation between the enhancement degree of contrast-enhanced ultrasound (CEUS) and the expression of CD147 and MMP-9 in carotid atherosclerotic plaques in patients with carotid endarterectomy and evaluate the diagnostic efficacy of CEUS using pathological results as the gold standard. Thirty-eight patients who underwent carotid endarterectomy (CEA) for carotid stenosis in the Department of Neurovascular Surgery of the Second People’s Hospital of Shenzhen from July 2019 to June 2020 were selected. Preoperatively, two-dimensional (2D) ultrasound scan was performed on all patients to assess the characteristics of the plaque and degree of stenosis, and CEUS was used to evaluate the surface morphology of the plaque and the distribution of neovascularization. Postoperatively, pathological sections and immunohistochemical analysis of CD147 and MMP-9 levels in the plaque were performed on the stripped plaque tissue, and the results were analyzed against the CEUS grading and pathological results. Among the 38 patients, pathological results showed that 10 and 28 were in the stable and vulnerable plaque groups, respectively. There were more smokers in the vulnerable plaque group than in the stable plaque group, with higher intraplaques CD147 and MMP-9. The difference in ultrasound plaque surface morphology grading and CEUS grading between the two groups was statistically significant. There was no significant difference in age, sex, incidence of complications such as hypertension, diabetes, and coronary heart disease between the two groups. CD147 was higher in the CEUS grade IV group than in the grades I (*P* = 0.040) and II (*P* = 0.010) groups. MMP-9 was higher in the CEUS grade IV group than in the grade II group (*P* = 0.017); MMP-9 was higher in the grade III group than in the grade II group (*P* = 0.015). Intraplaque contrast enhancement intensity was positively correlated with CD147 (*r* = 0.462, *P* = 0.003) and MMP-9 (*r* = 0.382, *P* = 0.018) levels. There was moderate consistency between the assessment of plaque vulnerability by 2D-ultrasound and by histopathological hematoxylin-eosin (HE) (kappa = 0.457, *P* > 0.05). 2D diagnosis of vulnerable plaque had a sensitivity of 85.7%, a specificity of 60.0%, a positive predictive value of 85.7%, a negative predictive value of 60.0%, and an accuracy of 78.0%. There was a strong consistency between the assessment of plaque vulnerability by CEUS and histopathological HE (kappa = 0.671, *P* < 0.01). CEUS had a sensitivity of 89.2%, a specificity of 80.0%, a positive predictive value of 92.6%, a negative predictive value of 72.7%, and an accuracy of 86.8% for the diagnosis of vulnerable plaques; CEUS is a reliable, non-invasive test that can show the distribution of neovascularization within vulnerable plaques, evaluate the vulnerability and risk of intraplaque hemorrhage, with a high consistency with pathological findings. The degree of intraplaque enhancement and the levels of CD147 and MMP-9 in the tissue were positively correlated.

## Introduction

Stroke is the second most common cause of mortality worldwide (Benjamin et al., 2017), with about 70% of cases with acute ischemic stroke and 25% with ischemic strokes caused by vulnerable carotid plaques (Magge et al., 2013; Wang W. et al., 2017). Previous studies have shown that intraplaque neovascularization (IPN) is significantly associated with plaque vulnerability and is the most powerful independent predictor of plaque rupture and hemorrhage (Saito et al., 2014). Contrast-enhanced ultrasound (CEUS) provides real-time dynamic visualization of IPN distribution and density, clearly demonstrates plaque contour boundaries, and provides a more comprehensive assessment of plaque stability.

Extensive data demonstrate the critical role of leukocyte differentiation antigen 147 (cluster of differentiation, CD147) in the complex process of atherogenesis, progression, and thrombosis (Joghetaei et al., 2013). As an extracellular matrix metalloproteinase inducer (EMMPRIN), it induces matrix metalloproteinases (MMPs), including MMP-9, whose active expression degrades collagen fibers in the fibrous cap and leads to plaque instability, which is one of the main molecular mechanisms of plaque rupture (Loftus et al., 2000; Gough et al., 2006).

Clinical studies on CD147 and MMP-9 are currently limited to serum, and there is a lack of studies at home and abroad on the correlation between CEUS assessment of carotid plaque stability and intraplaque CD147 and its mediated MMP-9 expression levels. Accordingly, this study aims to evaluate the diagnostic efficacy of CEUS using pathological findings as the gold standard, and to investigate the correlation between the degree of CEUS enhancement and the levels of CD147 and MMP-9 in tissues of carotid atherosclerotic plaques in patients with CEA.

## Materials and Methods

### Study Subjects

Thirty-eight patients (34 males and 4 females), aged 56–86 years, (68.3 ± 9.5 years), who were scheduled for carotid endarterectomy in the Department of Neurovascular Surgery, Shenzhen Second People’s Hospital from July 2019 to June 2020, were enrolled in this study. patient characteristics is shown in Table 1.

**TABLE 1:** General clinical information.

	**n/−x ± s**	**ratio(%)**
Age,y	68.9 ± 9.3	
Sex (F/M)	34/4	89%/11%
Stroke/TIA	37	97%
Smoking status	17	45%
Diabetes mellitus	15	39%
Hypertension	26	68%
Coronary heart disease	6	16%
Hyperlipidemia	38	100%
**Surface appearance**		
Grade I	6	16%
Grade II	21	55%
Grade III	10	26%
Grade IV	1	2%
**Neovascularization**		
Grade I	5	13%
Grade II	6	16%
Grade III	17	45%
Grade IV	10	26%
CD147( × 10^–2^)	2.437 ± 1.747	
MMP-9( × 10^–2^)	2.078 ± 2.011	

The inclusion criteria included Digital subtraction angiography (DSA) examination findings suggestive of Benjamin et al. (2017): moderate lumen stenosis (stenosis rate 50%–69%) with stroke symptoms (Wang W. et al., 2017); moderate lumen stenosis (stenosis rate 50%–69%), without stroke symptoms, with imaging findings suggestive of vulnerable plaque (Magge et al., 2013); severe lumen stenosis (stenosis rate 70%–99%) and incomplete occlusion. The following patients were excluded (Benjamin et al., 2017): patients with non-atherosclerotic carotid stenosis, such as aortitis and iatrogenic stenosis (Wang W. et al., 2017); patients with severe systemic diseases such as cardiac, hepatic, and renal insufficiency, malignant tumor, or hematologic disease that could not tolerate the procedure (Magge et al., 2013); patients with incomplete specimen acquisition that could not be analyzed to assess the nature of the plaque; and (Saito et al., 2014) patients with inability to sign the informed consent.

All patients underwent carotid vascular ultrasonography and carotid CEUS within one week before surgery. The indications for CEA were as follows: (1) cerebral ischemia and carotid artery stenosis > 50%, (2) cerebral ischemia and carotid artery stenosis > 70%, and (3) severe combined cerebrovascular and cardiac disease.

All patients enrolled in this study signed a plaque CEUS informed consent form.

### Study Methods

#### Clinical Information

The basic clinical information of each patient, including age, sex, height, weight, history of hypertension, coronary heart disease, diabetes, smoking, hyperlipidemia, and statin drug use, was collected; neurological clinical manifestations and related signs were recorded, and patients were asked whether they had a history of cerebral infarction or transient ischemic attacks (TIA) within six months before admission. The neurological signs and symptoms included TIA, limb weakness, hemiparesis, impaired consciousness, impaired speech function, and blurred vision.

The patients’ relevant laboratory test results before CEA were collected, including total cholesterol levels and triglycerides.

#### Evaluation of Plaque Characteristics by Conventional Carotid Ultrasound and Contrast-Enhanced Ultrasound

A ultrasound instrument (Supersonic Imagine AixPlorer, Provence en Aix, France) with a 10-2-line array probe at 2–10 MHz, was used. The subject was placed in a supine position with full exposure of the neck, and the target plaque was recorded by scanning the lower neck bilaterally from the bifurcation of the common carotid artery to the proximal segment of the external and internal carotid arteries. The plaques were measured according to the Chinese guidelines for vascular ultrasonography in stroke, following the basic principles of localization, characterization, and quantification to describe the location, morphology, echogenicity, fibrous cap integrity, presence or absence of ulceration, and the presence of stenosis and to determine the degree of stenosis.

The content and score of routine ultrasound plaque vulnerability scoring were as follows: morphology (regular, 0 points; irregular, 1 point); echogenicity (uniformly hyperechoic: 0 points, predominantly hyperechoic: 1 point, predominantly hypoechoic: 2 points, uniformly hypoechoic: 3 points); fibrous cap (smooth and intact: 0 points, not smooth and incomplete: 1 point); fibrous cap (smooth and incomplete: 1 point); ulceration (absent: 0 points, present: 1 point); degree of stenosis (< 50%: 0 points, 50%–69%: 1 point, 70%–89%: 2 points, 90%–99%: 3 points). The plaques were scored according to the above criteria with plaques that scored ≤ 4 being characterized as stable plaques, and those > 4 as vulnerable plaques (Fabiani et al., 2020).

**FIGURE 1:**
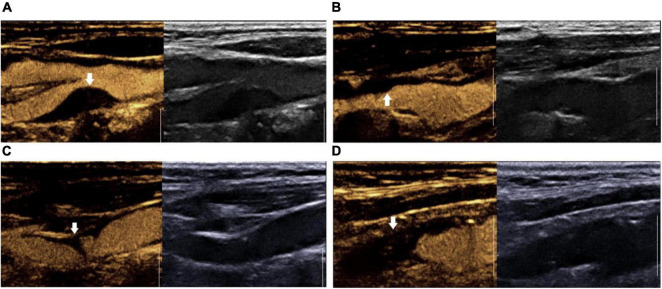
Surface morphology grading of contrast-enhanced ultrasound plaque. **(A)** Grade I: smooth plaque surface group; **(B)** Grade II: irregular plaque surface group. **(C)** Grade III: ulcerated plaque group; **(D)** Grade IV: carotid artery occlusion group.

The surface morphology of carotid plaques were characterized by CEUS in both long-axis and short-axis view. According to the surface morphology, plaques were classified into four groups: grade I, smooth plaque surface group; grade II, irregular plaque surface group; grade III, plaque ulcer formation group; and grade IV, carotid artery occlusion group (Jashari et al., 2016) (Figure 1).

**FIGURE 2:**
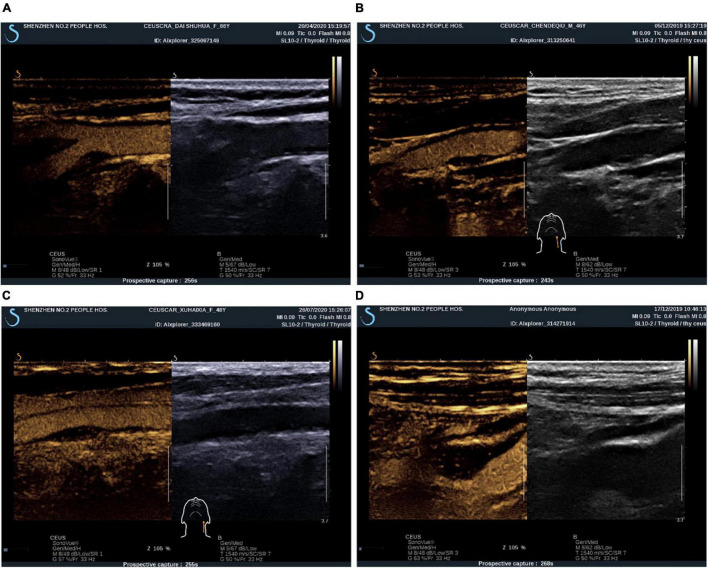
CEUS plaque enhancement grading. **(A)** Grade I: plaque without enhancement; **(B)** Grade II: plaque basal enhancement. **(C)** Grade III: plaque basal and shoulder enhancement; **(D)** Grade IV: plaque basal, shoulder and internal enhancement.

The procedure for carotid plaque CEUS is as follows: first, we switched to contrast mode, 1.5 ml contrast suspension (SonoVue, Bracca, Italy) was injected rapidly into the patient’s median elbow vein and flushed with 5 ml of saline. Intra-plaque contrast was observed for 5 min. During the examination, the probe was fan-shaped and oscillated minutely to observe the enhancement of all areas of the plaque as much as possible. The abovementioned process was repeated after the contrast had been completely excreted, if necessary, to continue the observation. The video clips were stored as raw data. Prior to the examination, all patients were required to give informed consent for CEUS and have their blood pressure measured. After the examination, the patient was allowed to leave after blood pressure was measured to ensure normalcy and sitting still for 15 min under observation with no adverse effects. After the contrast agent was injected, if dotted or short linear microbubble hyperechoes moved in a linear pattern around or inside the plaque, it indicated the formation of neovascularization within the plaque. Grading was performed based on the enhancement characteristics of carotid atherosclerotic plaque angiography, and semiquantitative analysis of neovascularization was performed (Saha et al., 2016): Grade I, no significant enhancement within the plaque; Grade II, enhancement at the base of the plaque; Grade III, enhancement at the base and shoulder of the plaque; and Grade IV, enhancement at the base, shoulder, and center of the plaque (Figure 2). The results of grading according to the above criteria were used for ultrasound staging of carotid atherosclerotic plaque susceptibility according to the plaque surface morphology and microvessel grading: grades I and II were stable plaques, and grades III and IV were vulnerable plaques (Zhao et al., 2018). When the plaque morphology and contrast enhancement grading of the same patient differed, the higher grade was considered.

#### Pathological Detection and Immunohistochemical Analysis

Fresh tissues were collected and fixed in 10% neutral buffered formalin for 12–24 h. After dehydration and immersion in wax, the tissues were embedded in paraffin and cross-sectionally sectioned 4 μm thick for hematoxylin-eosin (HE) staining; the pathologists were unaware of the ultrasonographic findings. Histopathological HE diagnostic criteria for vulnerable carotid plaques (Naghavi et al., 2003) are as follows: (i). Primary conditions [(i) thin plaque fibrous cap and presence of a large eccentric lipid core; (ii) presence of active inflammation within the plaque, that is, the presence of macrophages, monocytes, or T-cell infiltration; (iii) pathologic confirmation of > 90% carotid stenosis caused by the plaque; (iv) microscopic confirmation of a lacunar-like plaque]; (ii) Secondary conditions, such as if there was: ① obvious nodular calcification on the surface; ② bleeding within the plaque; ③ HE staining showing a bright yellow colored plaque; ④ disturbance of endothelial function of the vessel; ⑤ positive remodeling of the vessel wall. The presence of one major condition or two minor conditions was defined as a vulnerable plaque, while the opposite was defined as a stable plaque.

For immunohistochemical (IHC) staining, paraffin sections were dewaxed in water, followed by antigen repair, and placed in 3% hydrogen peroxide solution. Next, antigen repair was performed with 3% bovine serum albumin and incubated with primary and secondary antibodies, then diaminobenzidine chromogenic solution for color development and hematoxylin staining solution for re-staining nuclei were applied, and finally, they were dehydrated and sealed. All sections were scanned with 3D HISTECH Pannoramic (3D HISTECH, Hungary), and intra-tissue CD147 and MMP-9 were measured as follows: a patchy area was selected for each section, and the experimenter randomly selected at least four or more 400 × field-of-view maps, followed by screenshots. The screenshots were required to have tissue distribution over the entire field of view as much as possible, and the backlighting had to be consistent in each screenshot. Applying Image-Pro Plus 6.0 software, the criterion for judging all positive screenshots was that the microscopic display was the same tan color, and finally, each photo screenshot was analyzed and calculated to derive its positive cumulative optical density value and the area of tissue pixels within the glass section. The face density was calculated based on the above measurement data, and the formula was as follows: face density = cumulative optical density value/pixel area; the larger the face density value, the higher the level of positive expression in the tissue sections.

### Statistical Analysis

Statistical software (SPSS 26.0) was used, and the measurement data were expressed as x ± s, while the count data were expressed as percentages. Independent sample *t*-test, Fisher’s exact probability method, and Mann-Whitney test were used for comparison between groups. Comparisons between multiple groups were performed using the analysis of variance. Correlations between parameters were analyzed using Spearman’s correlation analysis. Ultrasonography and histopathological HE assessment of vulnerable plaques were assessed using Cohen’s k test, where kappa values of 0.41 to 0.60 indicated moderate agreement, 0.61 to 0.80 indicated a strong agreement, and 0. 81 to 1.00 indicated the strongest agreement. Differences were considered statistically significant at *P* < 0.05.

## Results

### General Clinical Data

**FIGURE 3:**
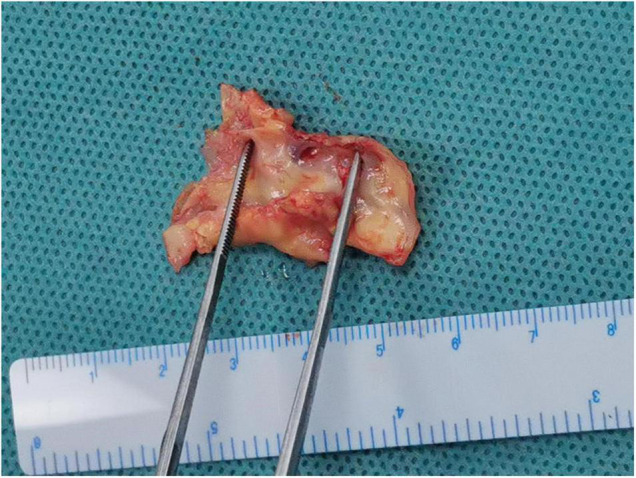
Gross carotid endarterectomy pathology specimen.

All 38 patients underwent carotid endarterectomy (see Figure 3 for gross pathological specimens) and carotid plaque CEUS; patient characteristics are shown in Table 1. Most (97%) of the patients had a history of cerebral infarction or TIA.

### Intergroup Comparison Between Stable and Vulnerable Plaque Groups

**FIGURE 4:**
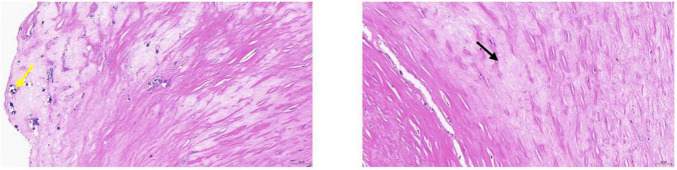
Pathological tissue showing stable plaque. Pathological HE and serial pathological sections showed a large amount of vascular smooth muscle cell edema with cell swelling in the tissue (black arrow); a few foci of calcification were seen locally (yellow arrow). CEUS presentation of this patient was grade II.

**FIGURE 5:**
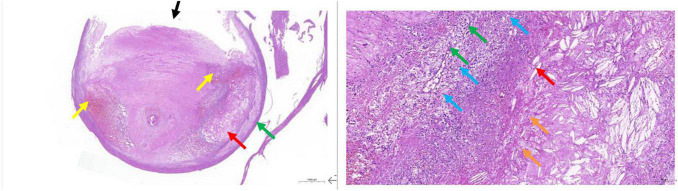
Pathological tissue showing vulnerable plaque. A large plaque with a small lumen is seen in the lumen of the vessel. The plaque contains mainly fibrous tissue (black arrow), the local structure is relatively loose, a large number of prismatic cholesterol crystals (red arrow), local hemorrhage (yellow arrow), and more new capillaries (green arrow), inflammatory cell infiltration mainly by foam cells (blue arrow), and a small amount of necrotic debris (orange arrow) are also seen.

According to histopathological HE results, patients were divided into the stable (10 patients) and vulnerable (28 patients) plaque groups (Figures 4, 5). It was found that the vulnerable plaque group included more smokers and higher CD147 and MMP-9 within the vulnerable plaques, than the stable plaque group; there were statistically significant differences in plaque surface morphology and CEUS gradings between the two groups. There were no statistically significant differences in age, sex, and whether complications including hypertension, diabetes, and coronary artery disease were present, between the two groups (Table 2).

**TABLE 2:** Comparison of general information on histopathological groupings.

**Characteristic**	**Stable plaque group (*n* = 10)**	**Vulnerable plaque group (*n* = 28)**	**Test value**	***P* value**
Age,y	68.6 ± 12.6	68.1 ± 8.3	0.129	0.898
Sex (M)	10	24		0.556
Smoking status	1	16		0.012
Hypertension	5	21		0.235
Diabetes mellitus	2	13		0.259
Coronary heart disease	1	3		1.000
Hyperlipidemia	10	28		
**Surface appearance**			−2.427	0.015
Grade I	3	3		
Grade II	4	17		
Grade III	0	10		
Grade IV	0	1		
**Neovascularization**			−2.598	0.013
Grade I	3	2		
Grade II	5	1		
Grade III	1	16		
Grade IV	2	8		
CD147 ( × 10^–2^)	1.075 ± 1.114	2.924 ± 1.684	−3.213	0.003
MMP-9 ( × 10^–2^)	0.588 ± 1.003	2.609 ± 2.022		0.002*

*Non-normally distributed, using independent sample rank sum test.

### Inter-Group Comparison of Ultrasonographic Grading

**FIGURE 6:**
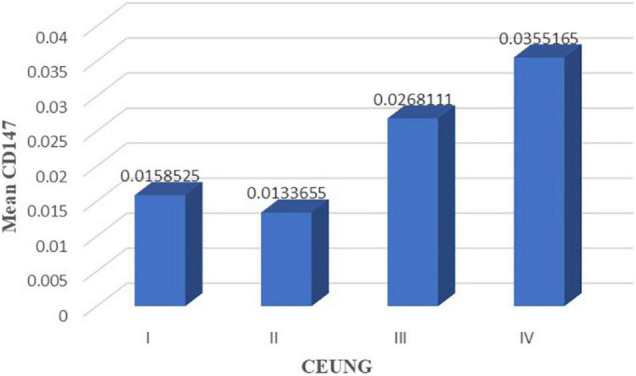
CD147 content in each group of CEUS neovascularization grading. CEUNG: Contrast-enhanced ultrasound neovascularization grading.

Comparison of CD147 content in pathological tissues between CEUS groups: grade IV group had higher CD147 in tissues than grade I group (*P* = 0.040) and grade II group (*P* = 0.010). However, there were no statistically significant differences in intra-tissue CD147 between the grades IV and III groups (*P* = 0.217), grades I and II groups (*P* = 789), grades I and III groups (*P* = 0.191), and grades II and III groups (*P* = 0.060) (Figure 6 and Table 3).

**TABLE 3:** Comparison of CD147 and MMP-9 among different groups in CEUS grade.

**CEUS grading**	**Number of cases**	**Age**	**CD147 (× 10** ^–^ **^2^)**	**MMP-9 (× 10** ^–^ **^2^)**
Grade I	5	68.2 ± 12.4	1.585 ± 1.363^Δ^	1.252 ± 1.237
Grade II	6	67.5 ± 12.2	1.337 ± 0.891	0.581 ± 0.844^†^
Grade III	17	66.9 ± 7.6	2.681 ± 1.820	2.633 ± 0.1.976*
Grade IV	10	72.0 ± 9.1	3.557 ± 1.790#**	2.909 ± 2.506*

Compared with the class I group, #*P* < 0.05; compared with the class II group, *P < 0.05 and **P < 0.01; compared with the class III group, †*P* < 0.05; compared with the class IV group, ΔP < 0.05.

**FIGURE 7:**
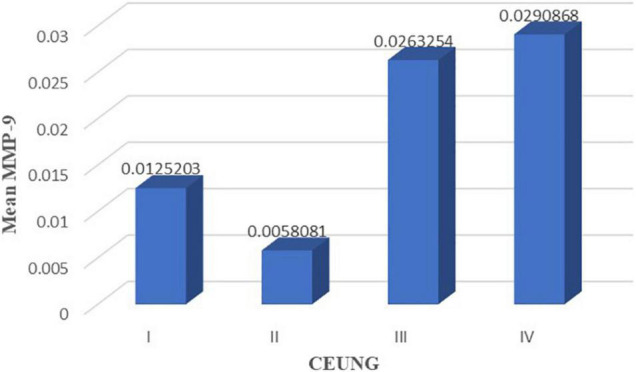
MMP-9 content in each group of CEUS neovascularization grading. CEUNG: Contrast-enhanced ultrasound neovascularization grading.

Comparison of intra-tissue MMP-9 levels between the CEUS groups: intra-tissue MMP-9 was higher in the grade IV group than in the grade II group (*P* = 0.017); intra-tissue MMP-9 was higher in the grade III group than in the grade II group (*P* = 0.015). However, there were no statistically significant differences in intra-tissue MMP-9 between grades I and II groups (*P* = 0.531), grades I and III (*P* = 0.154) groups, grade I and IV (*P* = 0.127) groups, and grades III and IV groups (*P* = 0.731) (Figure 7 and Table 3).

### Correlation Analysis of Intraplaque CD147 and MMP-9 Content With Ultrasonographic Neovascularization Grading

**FIGURE 8:**
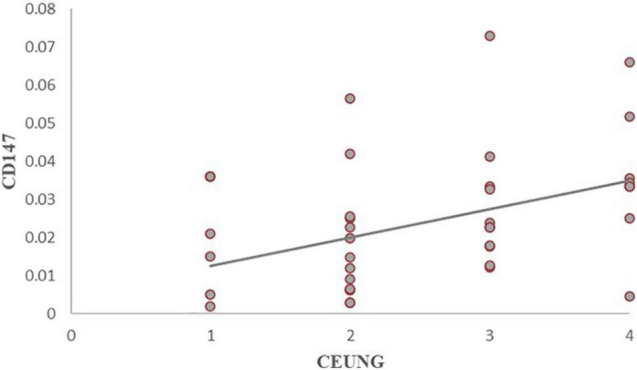
Scatter plot of CD147 content and CEUS neovascularization grading. CEUNG: Contrast-enhanced ultrasound neovascularization grading.

**FIGURE 9:**
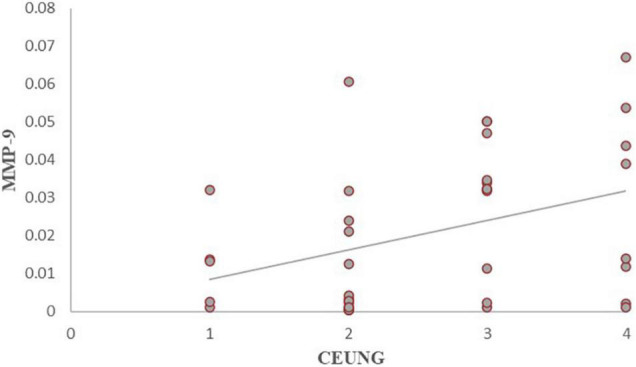
Scatter plot of MMP-9 content and CEUS neovascularization grading. CEUNG: Contrast-enhanced ultrasound neovascularization grading.

The results showed that CD147 content was positively correlated with ultrasonographic neovascular grading, with a statistically significant difference (*r* = histopathological 0.462, *P* = 0.003) (Figure 8). MMP-9 content was positively correlated with ultrasonographic neovascular grading, with a statistically significant difference (*r* = 0.382, *P* = 0.018) (Figure 9). However, the correlations were weak.

### Histopathological and 2D-Ultrasound and Ultrasonographic Assessment of Plaque Vulnerability Consistency Test

To evaluate the accuracy of CEUS in assessing plaque vulnerability, the results of CEUS were tested for concordance with pathological evaluation of plaque vulnerability using histopathology as the gold standard (Table 4), and the concordance between assessment of plaque susceptibility using CEUS and histopathological HE was strong (kappa = 0. 671, *P* < 0. 01). CEUS had high diagnostic efficacy in diagnosing vulnerable plaques, with a sensitivity of 89.2%, specificity of 80.0%, positive predictive value of 92.6%, negative predictive value of 72.7%, and accuracy of 86.8%.

**TABLE 4:** Histopathological hematoxylin-eosin staining and CEUS assessment of plaque vulnerability consistency test.

**Histology**	**CEUS**		**Total**
	**Stable plaque**	**Vulnerable plaque**	
Stable plaque	8	2	10
Vulnerable plaque	3	25	28
Total	11	27	38

Meanwhile, the 2D-ultrasound scoring results were tested for agreement with pathological evaluation of plaque vulnerability using histopathology as the gold standard (Table 5), and the agreement between the 2D-ultrasound assessment of plaque vulnerability and histopathological HE assessment of plaque vulnerability was moderate (Kappa = 0. 457, *P* > 0. 05). The sensitivity of 2D-ultrasound in diagnosing vulnerable plaques was 85.7%, specificity 60%, positive predictive value 78%, negative predictive value 85.7%, and accuracy 60%. The results indicated that the accuracy of 2D-ultrasound in diagnosing vulnerable plaques was slightly lower than that of CEUS.

**TABLE 5:** Histopathological hematoxylin-eosin staining and two-dimensional ultrasound assessment of plaque vulnerability agreement test.

**Histology**	**2D ultrasonography (n)**		**Total**
	**Stable plaque**	**Vulnerable plaque**	
Stable plaque	6	4	10
Vulnerable plaque	4	24	28
Total	10	28	38

## Discussion

In recent years, the characteristics of atherosclerotic plaques have been extensively studied, and vulnerable plaque pathology includes active inflammation, thin fibrous cap with large eccentric lipid core, plaque rupture thrombosis, fibrous cap rupture ulcer formation, and severe luminal stenosis (> 90%) (Naghavi et al., 2003). Intraplaque neovascularization is associated with local intraplaque inflammation and suggests a higher risk of plaque bleeding and rupture (Michel et al., 2011). Contrast-enhanced ultrasound can identify the plaque morphology and IPN, which are important in the risk assessment of patients with atherosclerosis and in clinical decision making.

Recognized predisposing factors for stroke include age, sex, obesity, history of smoking, hypertension, diabetes, high LDL cholesterol levels, stroke, atrial fibrillation, and valvular disease (Wang C. et al., 2017; Thorsson et al., 2018). In a study including 1209 participants (450 smokers and 759 non-smokers) (Kiriyama et al., 2020), the incidence of carotid plaque hypoechogenicity and ulceration was significantly higher in smokers than in non-smokers. The results of this study showed a significant difference in smoking history between the two groups of patients (stable and vulnerable plaques groups). This is similar to the results of previous studies. Age, sex, hypertension, diabetes mellitus, and history of coronary artery disease did not differ statistically between the two groups and were not consistent with established clinical perceptions. This may be related to the small sample size of the cases in this group.

The concept of neovascular involvement in the pathophysiology of atherosclerosis dates back to the discovery by Köester in 1876 and Winternitz in 1938 that atherosclerotic segments of coronary arteries have a rich network of vessels that extend from the outer membrane of the artery through the entirety of the intima. Intraplaque hypoxia and local inflammation trigger intraplaque neovascularization. These vessels are devoid of smooth muscle cells and simply consist of endothelial cells (Doran et al., 2008). Pathological neovascularization results in vessels that lack connective tissue and basement membrane, which make them more brittle and permeable, leading to lipid deposition and intraplaque hemorrhage (IPH). Intraplaque hemorrhage is the primary source of lipid nuclear expansion. Plaques with IPH are associated with rapid disease progression, recurrent bleeding, and other clinical events (Camare et al., 2017; Mura et al., 2020).

Conventional ultrasound is the most commonly used non-invasive diagnostic method for assessing the severity of atherosclerosis in clinical practice. Conventional ultrasound indicates the composition of the intraplaque components through a grayscale display with limitations in the detection of ulcerated plaques and intraplaque neovascularization (Schinkel et al., 2020). The study by Toshiyasu Ogata showed that the percentage of patients histologically classified with plaque rupture showed a significantly higher ulcerated appearance with CEUS than with conventional ultrasound (Hamada et al., 2016). Ulceration is one of the important features of vulnerable plaques. CEUS clearly outlines the luminal morphology and better demonstrates ulcerated plaques.

Currently, CT, MRI, and CEUS can be used to evaluate intraplaque neovascularization. Because of the small diameter of the new vessels and motion artifacts, CT and MRI are difficult to perform; the contrast agent used can cause hepatotoxicity, renal toxicity, and allergic reactions, while CEUS can be used to observe microbubble filling in plaque neovascularization in real time, with microbubbles as contrast agents metabolized mainly by respiration, which is much safer (Saba et al., 2019). A large number of studies has confirmed that the degree of contrast enhancement of plaques correlates well with the microvasculardensity (MVD) of plaques with good specificity (Vavuranakis et al., 2013; Li et al., 2014). Studies have shown that the inflow of small emboli into the blood is positively correlated with neovascularization in plaques (Ritter et al., 2013). In this study, ultrasound was performed using conventionally and with contrast enhancement. In this study, plaque stability was assessed using conventional ultrasound and contrast-enhanced ultrasound, and histopathological findings were used as controls. The accuracy of CEUS in assessing vulnerable plaques was higher than that of conventional ultrasound, confirming the value of CEUS in assessing carotid plaque stability.

The exact mechanism of plaque rupture is unknown, but excessive degradation of the extracellular matrix scaffold by MMPs is considered as one of the main molecular mechanisms involved (Dollery and Libby, 2006). The structural integrity of the plaque’s fibrous cap and the tolerance of external forces are mainly mediated by the extracellular matrix. MMP-9 is one of the main mediators of extracellular matrix degradation. Clinical as well as animal studies have confirmed that MMP-9 levels are increased in both peripheral blood and plaques in atherosclerosis, and the degree of increase is positively correlated with lesion severity (Lindsey et al., 2001; Cho and Reidy, 2002). CD147 is a widely distributed transmembrane protein, also known as EMMPRIN, which induces MMP-9 expression and is a key mediator of inflammatory and immune responses; it has been correlated with intraplaque angiogenesis (Chen et al., 2009). According to Seizer et al. (2010) silencing CD147 with the siRNA gene effectively reduced the upregulation of monocyte-derived foam cells expressing MMP-9 and MTl-MMP, reducing matrix degradation and playing an important role in plaque stabilization. Xu et al. (2018) showed that patients with TIA and acute cerebral infarction had significantly higher serum CD147 levels; increased CD147 levels in vulnerable plaques compared with those in stable plaques, and serum CD147 levels were associated with increased risk of stroke following episodes of TIA. In this study, the expression of CD147 and MMP-9 factors in carotid plaques was examined at the molecular level, and the levels of both factors were higher in the histopathologically vulnerable plaque group than in the stable plaque group, which is consistent with previous studies. The levels of CD147 and MMP-9 were higher in the histopathologically vulnerable plaque group than in the stable plaque group. The stronger plaque CEUS enhancement represented more neovascularization, and neovascularization-rich plaques were more susceptible to bleeding and inflammatory responses and had higher intra-tissue CD147 and MMP-9 levels.

This study has several limitations. To obtain intraplaque rather than serum levels of CD147 and MMP-9, this study was conducted in patients who underwent carotid endarterectomy, and the sample size was relatively small due to the limited study time and the impact of the novel coronavirus epidemic. The assessment of plaque stability by CEUS was also limited to patients who underwent carotid endarterectomy and could not be extrapolated to patients with carotid atherosclerotic plaques in general. Second, the use of lipid-lowering drugs may lead to a biased selection of study subjects, affecting the analysis of risk factors and possibly affecting the CD147 levels within the plaque. In addition, the method used for the evaluation of CEUS results was semi-quantitative due to equipment and software limitations, and a quantitative study could not be performed. In the future, it will be necessary to continue to accumulate more cases and select a more accurate quantitative evaluation method to obtain more reliable results.

## Conclusion

Contrast-enhanced ultrasound (CEUS) can show the amount of neovascularization within the plaque, indicate the risk of bleeding within the plaque, determine the vulnerability of the plaque, and has a high level of agreement with pathological findings; it is a reliable and non-invasive examination method for evaluating vulnerable plaques. The higher the degree of plaque enhancement, the higher the levels of CD147 and MMP-9 in the tissue.

## Data Availability Statement

The original contributions presented in the study are included in the article/supplementary material, further inquiries can be directed to the corresponding author.

## Ethics Statement

The studies involving human participants were reviewed and approved by the Ethics Committee of the Second People’s Hospital of Shenzhen. The patients/participants provided their written informed consent to participate in this study.

## Author Contributions

ZL, SH, and XW contributed to conception and design of the study. ZL, SH, XW, YH, and JW organized the database. ZL and SH performed the statistical analysis. SH wrote the first draft of the manuscript. ZL, SH, and LZ wrote sections of the manuscript. All authors contributed to manuscript revision, read, and approved the submitted version.

## Conflict of Interest

The authors declare that the research was conducted in the absence of any commercial or financial relationships that could be construed as a potential conflict of interest.

## Publisher’s Note

All claims expressed in this article are solely those of the authors and do not necessarily represent those of their affiliated organizations, or those of the publisher, the editors and the reviewers. Any product that may be evaluated in this article, or claim that may be made by its manufacturer, is not guaranteed or endorsed by the publisher.
